# Hand Hygiene Compliance and Associated Factors among Healthcare Workers in Ethiopia: A Systematic Review and Meta-Analysis

**DOI:** 10.1155/2021/7235248

**Published:** 2021-12-21

**Authors:** Negasa Eshete Soboksa, Belay Negassa, GirumGebremeskel Kanno, Zemachu Ashuro, DinkineshBegna Gudeta

**Affiliations:** ^1^Department of Environmental Health, College of Health Sciences and Medicine, Dilla University, Dilla, Ethiopia; ^2^College of Health and Medical Sciences, Arsi University, Asallaa, Ethiopia

## Abstract

**Background:**

Promoting hand hygiene compliance should be a priority for health authorities and all healthcare facilities at all levels. Therefore, this systematic review and meta-analysis aimed to provide a pooled estimate of hand hygiene compliance and associated factors among healthcare professionals in Ethiopia.

**Methods:**

PubMed, Science Direct, EMBASE, the Google search engine, and Google Scholar were used to retrieve studies that were eligible for the study. The searches included all studies published in English prior to July 2021. Using a structured data extraction format, two authors independently extracted the required data. STATA Version 16 software has been used for statistical analysis. To measure the heterogeneity of the studies, the Cochrane Q-test statistics and *I*2 test were used. Because of the significant heterogeneity, a random-effects model was used.

**Results:**

The pooled hand hygiene compliance among healthcare workers in Ethiopia was 38% (95% CI: 0.16–0.59). According to the study's subgroup analysis, Addis Ababa City administration health workers had the highest hand hygiene compliance, at 73% (95% CI: 0.50–0.96), while SNNP regional state had the lowest, at 9% (95% CI: 0.05–0.13). Presence of hand hygiene promotion (OR: 2.14, 95% CI: 1.04–3.24), towel/tissue paper availability (OR: 3.97, 95% CI: 2.09–5.86), having a positive attitude toward hand hygiene (OR: 1.79, 95% CI: 1.28–2.30), having good knowledge about hand hygiene (OR: 3.45, 95% CI: 1.26–5.64), and being trained for hand hygiene (OR:4.97, 95% CI:1.81–8.14) were significantly associated with hand hygiene compliance.

**Conclusion:**

In this analysis, hand hygiene compliance among healthcare workers in Ethiopia was less than half. Providing hand hygiene promotion, towel/tissue paper presence, having a positive attitude toward hand hygiene, having good knowledge about hand hygiene, and being trained for hand hygiene were important variables for the increment of hand hygiene compliance.

## 1. Introduction

Hand hygiene is the act of either hand washing with soap and water or hand disinfection to eliminate viruses, bacteria, and other microorganisms, as well as dirt, grease, and other harmful and unwanted substances that have become attached to the hands [1, 2]. It has been shown to be highly effective in preventing/reducing the occurrence of health-related infections from advanced healthcare systems to primary healthcare settings [3, 4]. Hand hygiene should be practiced at five crucial points in health care: before contact with a patient, before an aseptic procedure, after contact with a patient, after contact with body fluids, and after touching a patient's surroundings, according to the World Health Organization [5].

A systematic review conducted by Thames Valley University revealed that there was strong evidence that direct patient contact resulted in pathogen contamination of hands of healthcare workers [6]. Healthcare workers' hands are the most common means of transmitting healthcare-associated pathogens from patient to patient and within the healthcare setting [7]. A study has showed that roughly half of all healthcare-associated infections are caused by the hands of healthcare providers [8]. Hence, adherence to hand hygiene is an important infection control practice for reducing healthcare-associated infections [5].

Healthcare-associated infections and poor hand hygiene compliance among healthcare workers have a bigger impact on patients in healthcare settings [9]. Hands are contaminated with a microorganism during patient care unless prescribed hand hygiene compliance of health-care providers is followed [10]. In addition to each health provider's particular responsibilities, promoting hand hygiene compliance should be a priority for health authorities and all healthcare facilities at all levels [5]. However, several studies have shown considerable variations in hand hygiene compliance among healthcare providers prior to patient engagement. According to Erasmus et al.'s review analysis, all healthcare workers had a 21% compliance rate with hand hygiene before patient contact. Compliance after patient contact, on the other hand, was higher, with a median compliance rate of 47% [9].

Hand hygiene compliance is the most critical factor in preventing and controlling the spread of healthcare-associated illnesses; nevertheless, hand hygiene compliance remains low over the world [11]. In Ethiopia, hand hygiene compliance among healthcare workers varies from health facility to health facility or from regional state to regional state [12–21]. Hand hygiene compliance among healthcare workers in the country ranges from 9.2 to 89.5%, according to study findings [15, 18], and the factors associated with hand hygiene compliance have been inconsistent [12–21].

Even though disparities in hand hygiene compliance and associated factors exist in Ethiopian healthcare facilities, they have not been thoroughly investigated. Thus, the objective of this study was to give a pooled estimate of hand hygiene compliance and associated factors among healthcare workers in Ethiopia. This study focuses on hospitals because the risk of receiving and transferring infection is greatest there. The following research questions were addressed in this study: (1) What is the level of hand hygiene compliance among Ethiopian healthcare workers? (2) What factors influence hand hygiene compliance among Ethiopian healthcare workers? The study's findings may assist the governmental and nongovernmental organizations, as well as other stakeholders, in developing and implementing effective infection prevention methods in healthcare settings.

## 2. Materials and Methods

### 2.1. Study Design and Reporting

A systematic review and meta-analysis were carried out to provide a pooled estimate of hand hygiene compliance and associated factors among healthcare workers in Ethiopia. This meta-analysis was conducted according to the Preferred Reporting Items for Systematic Reviews and Meta-Analyses Guideline.

### 2.2. Eligibility Criteria

The review included only observational studies (cross-sectional studies, case-control studies, and cohort studies) on healthcare providers working in Ethiopian health facilities that reported hand hygiene compliance and associated factors. Furthermore, articles written in English that had previously been published, as well as those that had not yet been published, were included, regardless of publication year. Articles that were inaccessible despite at least two e-mail contacts with the primary authors were, however, excluded. The exclusion of these articles was due to an inability to determine the articles' content in the absence of a complete text. Excluded were studies in which it was difficult to extract the necessary information.

### 2.3. Information Sources and Search Strategy

Two reviewers (NES and DBG) searched independently for articles, which were available online before July 2021, from PubMed, Science Direct, EMBASE, Google search engine, Google Scholar, and references of other studies. To obtain the articles, the search used the following MeSH and free-text terms: “hand hygiene,” “hand disinfection,” “hand washing,” “compliance,” “guideline adherence,” “health personnel,” “healthcare providers,” and “Ethiopia.” Boolean operators (AND/OR) were used to combine the terms. The full electronic search strategy for PubMed is shown online (Table S1). Preferred Reporting Items for Systematic Reviews and Meta-Analyses (PRISMA) tool was applied to conduct this systematic review and meta-analysis [22].

### 2.4. Selection Process

Following the inclusion and exclusion criteria principles, the studies were established separately by two reviewers (DBG and NES). Based on the importance of their titles and abstracts, the studies were chosen first. Next, to validate eligibility, full-text articles were collected and checked. In discussions with the primary author to reach an agreement, any contradictions were resolved. Discrepancies are overcome or determined by consensus by a third reviewer (BN).

### 2.5. Data Collection Process

The studies retrieved from different databases were exported into Mendeley Desktop Reference Management software version 1.19.5 (Mendeley Ltd., Elsevier, Netherlands) and then duplicates were excluded. To summarize the analysis collection methods, the PRISMA flow diagram was used.

### 2.6. Data Items

The following information was extracted: name of the first author or research group, year of publication, region/health facility, study design, sample size, and status of hand hygiene compliance of healthcare workers. Data on factors associated with hand hygiene compliance in working wards was also independently collected by reviewers. For the second outcome, data were extracted in the form of two-by-two tables, and the odds ratio (OR) was calculated using the original studies' findings.

### 2.7. Study Risk of Bias Assessment

Two authors independently assess the quality of each included study. To assess the quality (risk of bias), we used the Hoy et al. (2012) tool for addressing internal and external validity using ten criteria [23]. The tool primarily included (1) population representation, (2) sampling frame, (3) methods of participant selection, (4) nonresponse bias, (5) data collection directly from subjects, (6) acceptability of case definition, (7) reliability and validity of study tools, (8) mode of data collection, (9) length of prevalence period, and (10) appropriateness of numerator and denominator. Each item was categorized as having either a low or a high bias risk. “Not clear” was classified as having a high risk of bias. Finally, the overall bias risk score was then graded according to the number of high bias risk per study: low (≤2), moderate (3–4), and high (≥5).

### 2.8. Outcome of Interest

The primary outcome of this study was to determine the hand hygiene compliance of healthcare providers. Hand hygiene compliance is conformity to a rule, such as a specification, policy, standard, or regulation. It is a state of conformity to specified guidelines, specifications, or legislation. We included studies that reported compliance self-reported by healthcare workers and that was measured by direct observation according to WHO recommendations [24]. The second outcome of interest was to determine the factors that are associated with hand hygiene compliance among healthcare workers in Ethiopia. It was determined using the odds ratio (OR) and calculated based on binary outcomes from the included primary studies.

### 2.9. Data Analysis

Using Microsoft Excel spreadsheets, data were collected from each study and imported for analysis into STATA version 16 statistical software. A *p* ≤ 0.05 was identified as statistically significant. The *I*^2^ statistic was used to assess the heterogeneity among the studies analyzed [25]. An *I*^2^ > 50% or *p* < 0.1 indicated heterogeneity, for which the random-effects model was utilized. In addition, publication bias was evaluated through visual inspections of funnel plots and Egger's test, with a value of less than 0.05 as a cutoff point to declare the presence of publication bias. Moreover, to minimize random variations between the point estimates of the primary study, subgroup analysis was performed based on region/health facility, sample size, and study participant profession. A leave-one-out sensitivity analysis in hand hygiene compliance among health-care workers in Ethiopia was conducted to identify the potential source of heterogeneity in the analysis. In this study, the effect size is the pooled prevalence and odds ratio.

## 3. Results

### 3.1. Study Selection Process

**Figure 1:**
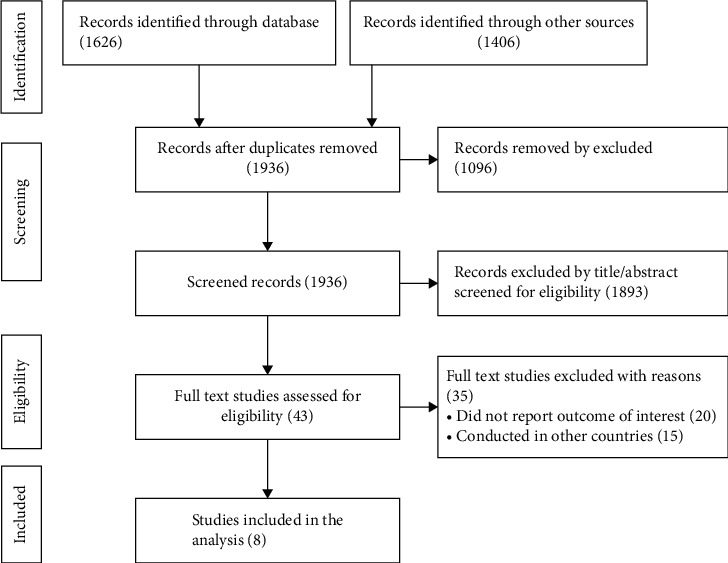
Flow diagram of included relevance studies identified by the systematic search strategy.

In our initial literature search, electronic databases and additional hand searches yielded a total of 3032 published and unpublished records. Due to overlap, 1096 records were removed. After reading the titles/abstracts, the 1920 records were excluded from the 1936 records. Then 43 records were screened for eligibility. Based on our research questions, 35 records were excluded due to their nonrelevance for this review. Finally, this meta-analysis included eight records (Figure 1).

### 3.2. Included Studies Description

As described in Table 1, the 8 cross-sectional studies conducted from 2014 to 2020 were included in this systematic review and meta-analysis. In this meta-analysis, the study participants of primary study studies were health-care professionals working in different wards. In the present meta-analysis, four Ethiopian regional states and one administrative town were represented. Specifically, three studies were from Amhara regional state [12–14], one study was from the Southern Nations, Nationalities, and Peoples Region (SNNP) [15], three studies were from Addis Ababa [16–18], and one study was from Harari regional state [19]. Regarding the level of hand hygiene compliance, it ranged from 9.20% to 89.5%. The lowest hand hygiene compliance of healthcare workers was reported in a study conducted in Wachemo University Hospital, SNNP [15], whereas the highest level was reported in a study conducted in Abet Hospital in Addis Ababa [18].

### 3.3. Risk of Bias

The quality of each original study was assessed using a risk of bias tool (Hoy et al. (2012)). Among the 8 included studies, 62.5% of the studies had a low risk of bias according to our assessment [12, 14, 16, 17, 19], while the remaining 37.5% of the included studies had a moderate risk of bias [13, 15, 18] (Table S2).

### 3.4. Hand Hygiene Compliance among Healthcare Professionals in Ethiopia

**Figure 2:**
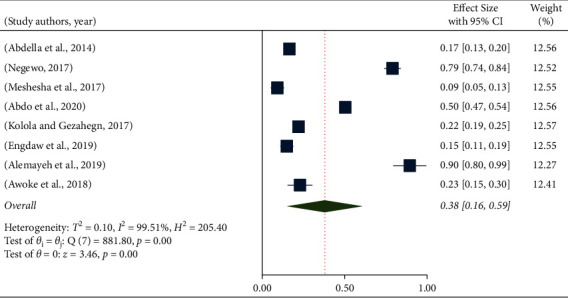
Forest plot of the pooled level of hand hygiene compliance among healthcare professionals in Ethiopia.

The pooled hand hygiene compliance among health workers in Ethiopia was 38% (95% CI: 0.16–0.59). The papers considered in this study have a high level of heterogeneity (*I*^2^ = 99.51, *p* = 0.001). Therefore, in order to determine the pooled level of hand hygiene compliance, a random effect meta-analysis model was used. According to this meta-analysis, the lowest hand hygiene compliance was reported from a study conducted in Wachemo University Hospital (9%), whereas the highest was reported by a study conducted in AaBET Hospital in Addis Ababa (90%) [15, 18] (Figure 2).

### 3.5. Subgroup Analysis

Subgroup analysis was performed based on the regional state/city administration of the country where the studies were conducted, the sample size, and the profession of study participants. Accordingly, Addis Ababa City administration health workers had the highest hand hygiene compliance at 73% (95% CI: 0.50–0.96) and Harari regional state also had the next hand hygiene compliance at 23% (95% CI: 0.13–0.30), while SNNP regional state had the lowest hand hygiene compliance at 9% (95% CI: 0.05–0.13). In terms of sample size, hand hygiene compliance of health workers was higher in studies with a sample size of 400, 43% (95% CI: 0.10–0.75) compared to studies with a sample size ≥400, 38% (95% CI: 0.09–0.50). In terms of subgroup analysis by profession type, studies involving only nurses reported higher hand hygiene compliance of 51% (95% CI: −0.04–1.06), compared to studies involving all types of healthcare workers (Table 2).

### 3.6. Publication Bias

**Figure 3:**
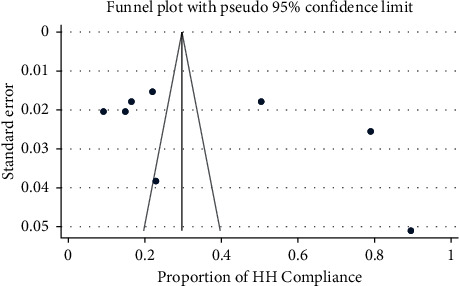
Funnel plot to assess publication bias among included studies. HH = hand hygiene.

Visual examination of the funnel plot showed asymmetric distribution of studies implies no publication bias (Figure 3). Furthermore, we used Egger's and Begg's tests to detect the presence of publication bias, and the results showed that there was no statistically significant publication bias in assessing the level of hand hygiene compliance among healthcare workers (*p* values of 0.07 and 0.0.53, respectively).

### 3.7. Sensitivity Analysis

Table 3 displays the sensitivity analysis of hand hygiene compliance for each study that was removed one at a time. To identify the potential source of heterogeneity in the analysis, a leave-one-out sensitivity analysis in hand hygiene compliance among healthcare workers in Ethiopia was performed. The findings revealed that no single study had an effect on the overall hand hygiene compliance of health-care professionals.

### 3.8. Factors Associated with Hand Hygiene Compliance

**Figure 4:**
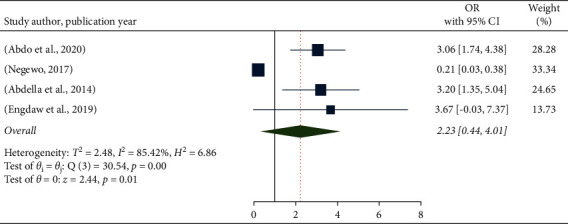
The pooled odds ratio of the hand hygiene compliance and availability of water and soap among health-care providers in Ethiopia.

In this meta-analysis, the association between hand hygiene compliance and the availability of water and soap in healthcare facilities was assessed using four studies [12, 14, 16, 17]. Study participants who worked in health facilities with adequate soap and water for hand washing were 2.23 times more likely to have good hand hygiene compliance than study participants who worked in health facilities with insufficient soap and water for hand washing (OR: 2.23, 95% CI: 0.44–4.01). However, the findings revealed no significant association and a high level of significant heterogeneity (85.42%) across the included studies (Figure 4).

**Figure 5:**
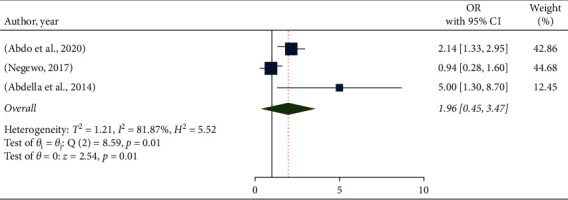
The pooled odds ratio of the association between hand hygiene compliance and knowing the functionality of infection prevention committee in Ethiopia.

The pooled odds ratio of hand hygiene compliance and knowing the functionality of the infection prevention committee in a health facility in Ethiopia was also computed using four studies [12, 14, 16, 17]. As a pooled result, study participants who knew the functionality of the infection prevention committee in a healthcare facility were 1.96 times more likely to have good hand hygiene compliance than those who did not (OR: 1.96, % CI: 0.45–3.47). However, the difference was not significant, and the included studies had a large amount of heterogeneity (Figure 5).

**Figure 6:**
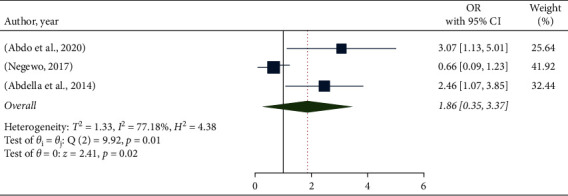
The pooled odds ratio of the association between hand hygiene compliance and functionality of sink in Ethiopia.

In this meta-analysis, the association between hand hygiene compliance and the functionality of sinks in healthcare facilities in Ethiopia was assessed using three studies [12, 16, 17]. According to the findings of these three studies, hand hygiene compliance was not significantly linked to the presence of a functional sink. As a result, when comparing healthcare employees working in facilities with functional sinks to their counterparts, the likelihood of good hand hygiene compliance was 1.86 times higher (OR: 1.86, 95% CI: 0.35–3.37) (Figure 6).

**Figure 7:**
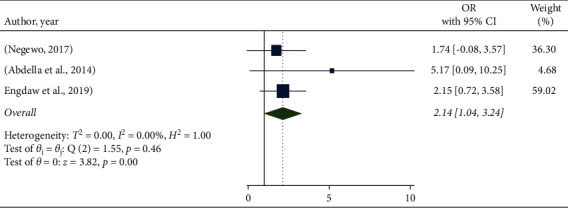
The pooled odds ratio of the association between hand hygiene compliance and hand hygiene promotion in health facility in Ethiopia.

Figure 7 shows that hand hygiene promotion in health facilities was found to be significantly associated with health-care personnel's hand hygiene compliance. Those who worked in a health facility where hand hygiene promotion was provided had 2.14 times the compliance of those who did not receive it (OR: 2.14, 95% CI: 1.04–3.24).

Table 4 also shows the pooled odds ratio of factors associated with hand hygiene compliance among health workers. The availability of towels or tissue paper, the presence of a hand hygiene protocol, attitude toward hand hygiene, and knowledge about hand hygiene all had a significant impact on healthcare professionals' hand hygiene compliance. Hand hygiene compliance was not significantly associated with the use of alcohol-based hand rubs. On the other hand, only studies included in the analysis of the association between the presence of alcohol-based hand rubs and hand hygiene compliance had a high level of heterogeneity. The others, on the other hand, were homogeneous or slightly heterogeneous. Hand hygiene compliance was 3.97 (OR: 3.97, 95% CI: 2.09–5.86) times higher among healthcare employees who worked in a facility that provided towels or tissue paper. Similarly, trained healthcare employees had 4.97 times more compliance than nontrained staff (OR: 4.97, 95% CI: 1.81–8.14) (Table 4).

## 4. Discussion

Hand hygiene compliance is the most critical factor in preventing and controlling the spread of healthcare-associated illnesses [11]. But hand hygiene compliance is low and varies from health facility to health facility or from region to region in Ethiopia [15, 18]. As a result, the aim of this review was to assess hand hygiene compliance and associated factors in Ethiopia by reviewing the findings of previous studies.

The pooled hand hygiene compliance among health-care workers in Ethiopia was 38% (95% CI: 0.16–0.59). The results of our study were substantially equivalent to those of a systematic review of general patient populations in industrialized countries (40%) [9]. Similarly, these findings are similar to those of another observational study conducted in Istanbul, Turkey, which found 37.0% hand hygiene compliance among health-care professionals, raising doubts that it was not a systematic review [26]. Despite the fact that the studies were not systematic reviews, the results were consistent with a study conducted in Ghana before and during the COVID-19 pandemic in selected primary hospitals (51%) [27], Tamale Teaching Hospital (49%) [28], and among exposed healthcare workers in COVID-19 treatment centers (97.5%) [29]. The findings contradicted a research undertaken by the University of Chicago Medical Center during the COVID-19 pandemic (compliance = 100%) [30]. The plausible explanation is that there was a high degree of infection prevention and control promotion (hand hygiene), as well as training for healthcare workers during the COVID-19 pandemic.

According to the study's subgroup analysis, Addis Ababa City administration health workers had the highest hand hygiene compliance at 73% (95% CI: 0.50–0.96), Harari regional state had the next highest hand hygiene compliance at 23% (95% CI: 0.13–0.30), and SNNP regional state had the lowest hand hygiene compliance at 9% (95% CI: 0.05–0.13) [15, 18, 19]. This variation could be explained by differences in sociodemographics, working environment setup, and the safety of the work, workload, and patient flow. Our study subgroup analysis by profession type showed that studies involving only nurses reported higher hand hygiene compliance of 51% (95% CI: −0.04–1.06), compared to studies involving all types of health-care workers. The finding was in line with a systematic review conducted by Erasmus et al., which reported that nurses had higher hand hygiene compliance than others [9]. The possible reason might be that, for the time, the situations that were associated with a higher compliance rate were those having to do with dirty tasks.

The aim of this study was also to find out what factors influence hand hygiene compliance among Ethiopian healthcare employees. Hand hygiene compliance was significantly associated with the presence of hand hygiene promotion in health facilities, towel/tissue paper availability, having a positive attitude toward hand hygiene, having good knowledge about hand hygiene, and being educated about hand hygiene. Hand hygiene compliance was 2.14 times higher among healthcare workers who worked in a facility that promoted hand hygiene compared to those who did not. Hand hygiene is a fundamental measure for reducing healthcare-associated infections. Promoting hand hygiene is important for everyone who works in a health-care facility to stay up to date on the importance of hand hygiene and its indications and to demonstrate the proper procedures for hand rubbing and hand washing.

In the present study, those who had a positive attitude toward hand hygiene were 1.79 times more likely to have good hand hygiene compliance than those who had a negative attitude toward hand hygiene. This was in agreement with a previous study conducted in Jordan, which found that healthcare providers' attitudes were strongly linked to high hand hygiene compliance [31]. This could be due to personal experience, the respondent's educational status, the presence of positive peer pressure, a positive professional attitude toward hand hygiene compliance, social factors, or religious institutions.

Hand hygiene compliance was also associated with knowledge about hand hygiene. Those with a good knowledge of hand hygiene were 3.45 times more likely to comply in this study than those with poor knowledge. This was confirmed by a study conducted in Saudi Arabia's Prince Sultan Military Medical City, which found that healthcare workers' knowledge was significantly associated with good hand hygiene compliance [32]. This could be linked to having a good understanding of hand hygiene compliance, which can help you comply with hand hygiene in the recommended manner, identify the benefits and drawbacks of hand hygiene compliance, and identify the route of healthcare-acquired infection transmission and how to avoid it.

Trained healthcare providers for hand hygiene were 4.97 times more likely to have good hand hygiene compliance than those who were not trained healthcare providers. The findings of this study are in agreement with those of previous studies [20, 33]. This could be because training increased healthcare providers' knowledge, which had a significant effect on hand hygiene compliance, and those who received training were expected to be role models for others in terms of practicing good hand hygiene practices for the identification of risk and benefits in the transmission of healthcare acquired infections and how to prevent people.

There are a few limitations in this study. To provide this nationally based review, only articles written in English were included. Most of the included articles' reports on hand hygiene compliance among healthcare workers were based on self-reporting without confirmation, and the results could be influenced by social desirability bias. Furthermore, because all the studies included in this review were cross-sectional, the outcome variable may be influenced by other confounding variables. Due to the small number of research included, this meta-analysis only included studies from a few areas and town administrations around the country, which may indicate underrepresentation.

## 5. Conclusion

The results of this systematic review and meta-analysis found that hand hygiene compliance of healthcare workers in Ethiopia was low. According to the subgroup study, health personnel in the Addis Ababa City administration had the highest hand hygiene compliance, while those in the SNNP regional state had the lowest. Studies including only nurses showed higher hand hygiene compliance when compared to studies involving all types of healthcare providers. Hand hygiene compliance was significantly associated with the presence of hand hygiene promotion in health facilities, towel/tissue paper availability, having a positive attitude toward hand hygiene, having good knowledge about hand hygiene, and being educated for hand hygiene. On the other hand, hand hygiene compliance was not significantly associated with the availability of water and soap in the health-care facility, functionality of the sink, knowledge of the infection prevention committee's functionality, or the existence of alcohol-based hand rubs. Therefore, based on our findings, we recommend that healthcare facilities increase hand hygiene promotion and supply towel/tissue paper, as well as infection prevention and control training to improve knowledge and attitude about hand hygiene.

## Figures and Tables

**Table 1:** Descriptive summary of studies included in the meta-analysis of the hand hygiene compliance and associated factors among healthcare workers in Ethiopia.

S. no.	Author, publication year	Region/health facility	Study design	Sample size	Level of HH compliance (%)
1	Abdella et al. [12]	Amhara/Gondar University Hospital	Cross-sectional	405	16.5
2	Negewo [16]	Addis Ababa/Black Lion Hospital	Cross-sectional	288	79.0
3	Meshesha et al. [15]	SNNP/Wachemo University Hospital	Cross-sectional	214	9.20
4	Abdo et al. [17]	Addis Ababa/general hospitals in Addis Ababa	Cross-sectional	651	50.4
5	Kolola and Gezahegn [13]	Amhara/Debre Berhan Referral Hospital	Cross-sectional	917	22.0
6	Engdaw et al. [14]	Amhara/Public Primary Hospitals in central Gondar zone	Cross-sectional	335	14.9
7	Alemayehu et al. [18]	Addis Ababa/AaBET Hospital	Cross-sectional	38	89.5
8	Awoke et al. [19]	Harari/Hiwot Fana Specialized Hospital	Cross-sectional	116	22.9

**Table 2:** Subgroup analysis compares the pooled level of hand hygiene compliance among healthcare workers in Ethiopia.

Variables	Subgroup	Number of studies included	Level of hand hygiene compliance (95% CI)	Heterogeneity across the studies	
				*I* ^2^ (%)	*p* value
Region	Amhara	3	0.18 (0.14–0.22)	78.10	0.01
	Addis Ababa	3	0.73 (0.50–0.96)	98.12	0.001
	SNNP	1	0.09 (0.05–0.13)	–	–
	Harari	1	0.23 (0.13–0.30)	–	–

Sample size	<400	5	0.43 (0.10–0.75)	99.49	0.001
	≥400	3	0.38 (0.09–0.50)	99.13	0.001

Profession	Nurses only	2	0.51 (−0.04–1.06)	99.33	0.001
	All types of healthcare workers	6	0.34 (0.09–0.58)	99.57	0.001

**Table 3:** Sensitivity analysis of level of hand hygiene compliance among healthcare providers in Ethiopia.

Study excluded	Level of HH compliance (%)	95% CI	*I* ^2^ (%)	*Q*-value	*p* value
Abdella et al. [12]	41	0.17–0.65	99.51	815.60	<0.001
Negewo [16]	32	0.11–0.53	99.43	470.72	<0.001
Meshesha et al. [15]	42	0.19–0.65	99.50	760.26	<0.001
Abdo et al. [17]	36	0.12–0.61	99.54	717.83	<0.001
Kolola and Gezahegn [13]	40	0.16–0.64	99.50	841.18	<0.001
Engdaw et al. [14]	41	0.18–0.65	99.53	813.94	<0.001
Alemayeh et al. [18]	31	0.12–0.49	99.34	733.50	<0.001
Awoke et al. [19]	40	0.16–0.64	99.61	872.88	<0.001

**Table 4:** The pooled odds ratio of the factors associated with hand hygiene compliance of healthcare workers in Ethiopia.

S. no.	Variable	Included studies	Or (95% CI)	*I* ^2^ (%)	*p* value
1	Availability of towel/tissue paper	[12, 16]	3.97 (2.09–5.86)	0.00	0.57
2	Presence of alcohol-based hand rubs	[12, 14, 16]	3.57 (−1.42–8.60)	73.84	0.02
3	Presence of hand hygiene protocol	[14, 17]	1.87 (0.96–2.78)	30.65	0.28
4	Attitude towards hand hygiene	[14, 17]	1.79 (1.28–2.30)	0.00	0.81
5	Knowledge about hand hygiene	[12, 14]	3.45 (1.26–5.64)	0.00	0.45
6	Trained for hand hygiene	[12, 14]	4.97 (1.81–8.14)	0.00	0.63

## Data Availability

All relevant data are within the manuscript.

## Abbreviations

MeSH Terms: Medical Subject Heading Terms
OR: Odds ratio
PRISMA: Preferred Reporting Items for Systematic Reviews and Meta-Analyses
SNNPR: Southern Nations, Nationalities, and Peoples Region
Tw: Text word
WHO: World Health Organization.

## Supplementary Materials

Supplementary Materials Table S1: search strategy for database and other web searches. Table S2: Risk of Bias Assessment Tool of Eligible Articles by using the Hoy 2012 tool.
