# Supplementary material for: Hand Hygiene Compliance and Associated Factors among Healthcare Workers in Ethiopia: A Systematic Review and Meta-Analysis
Source: Adv Prev Med. 2021 Dec 21;2021:7235248. doi: 10.1155/2021/7235248 (PMC8692043; doi:10.1155/2021/7235248)
Supplement: Supplementary Materials — Table S1: search strategy for database and other web searches. Table S2: Risk of Bias Assessment Tool of Eligible Articles by using the Hoy 2012 tool. [file 7235248.f1.zip › 7235248.f1/Table S1 Searching.docx]

Table S1:.Search strategy for database and other web searches

| S.NO | Databases | Searching terms | Number of studies |
| --- | --- | --- | --- |
|  | PubMed/Medline | (((("Hand hygiene"[MeSH Terms] OR hand hygiene [tw] OR "hand disinfection"[MeSH Terms] OR handwashing[tw])) AND ("Compliance"[MeSH Terms] OR compliance [tw] OR "guideline adherence"[MeSH Terms]) AND ("Health personnel"[MeSH Terms] OR health care providers [tw]))) AND ("Ethiopia"[MeSH Terms] OR Ethiopia[tw]) | 4 |
|  | Google scholar | "Hand hygiene" OR "hand disinfection" OR "hand washing" AND "Compliance" OR "guideline adherence" AND "Health personnel" OR "health care providers" AND "Ethiopia" | 1,070 |
|  | Science direct | "Hand hygiene" OR "hand disinfection" OR "hand washing" AND "Compliance" OR "guideline adherence" AND "Health personnel" OR "health care providers" AND "Ethiopia" | 1586 |
|  | Embase | (((("Hand hygiene"[MeSH Terms] OR hand hygiene [tw] OR "hand disinfection"[MeSH Terms] OR handwashing[tw])) AND ("Compliance"[MeSH Terms] OR compliance [tw] OR "guideline adherence"[MeSH Terms]) AND ("Health personnel"[MeSH Terms] OR health care providers [tw]))) AND ("Ethiopia"[MeSH Terms] OR Ethiopia[tw]) | 36 |
|  | Other sources |  | 336 |
|  | Total retrieved records |  | 9025 |
|  | Records included in the review |  | 8 |

MeSH Terms = Medical Subject Heading Terms, Tw= text word
