# Supplementary material for: Hand Hygiene Compliance and Associated Factors among Healthcare Workers in Ethiopia: A Systematic Review and Meta-Analysis
Source: Adv Prev Med. 2021 Dec 21;2021:7235248. doi: 10.1155/2021/7235248 (PMC8692043; doi:10.1155/2021/7235248)
Supplement: Supplementary Materials — Table S1: search strategy for database and other web searches. Table S2: Risk of Bias Assessment Tool of Eligible Articles by using the Hoy 2012 tool. [file 7235248.f1.zip › 7235248.f1/Table S2 risk bias assassment form.docx]

| Table S2: Risk of Bias assessment Tool of Eligible Articles by using the Hoy 2012 tool | | | | | | | | | | | | |
| --- | --- | --- | --- | --- | --- | --- | --- | --- | --- | --- | --- | --- |
| S.no | Study | Representation | Sampling | Random selection | Non response bias | Data collection | Case Definition | Reliability and validity of study tool | Method of data collection | Prevalence period | Numerator and denominator | Summary Assessment |
| 1 | (Abdella *et al*., 2014) | Low | Low | Low | Low | Low | Low | Low | Low | Low | Low | Low |
| 2 | (Negewo, 2017) | Low | Low | Low | Low | Low | Low | Low | Low | Low | Low | Low |
| 3 | (Meshesha et al., 2017) | Low | High | High | High | Low | High | High | Low | Low | Low | Moderate |
| 4 | (Abdo *et al.,* 2020) | Low | Low | High | High | Low | Low | Low | Low | High | Low | Moderate |
| 5 | (Kolola, 2017) | Low | High | Low | High | Low | High | High | Low | Low | Low | Moderate |
| 6 | (Engdaw *et al.,* 2019) | Low | Low | Low | Low | Low | Low | High | Low | Low | Low | Low |
| 7 | (Alemayehu *et al*., 2019) | High | Low | Low | Low | Low | High | Low | Low | Low | Low | Low |
| 8 | (Awoke *et al.,* 2018) | Low | Low | High | Low | Low | Low | Low | Low | Low | Low | Low |

| **Risk of bias assessment tool: Yes (low risk); No (high risk)** | | |
| --- | --- | --- |
|  | 1. Representation: Was the study population a close representation of the national population | |
|  | 2. Sampling: Was the sampling frame a true or close representation of the target population? | |
|  | 3. Random selection: Was some form of random selection used to select the sample OR was a census undertaken? | |
|  | 4. Non-response bias: Was the likelihood of non-response bias minimal? | |
|  | 5. Data collection: Were data collected directly from the subjects? | |
|  | 6. Case definition: Was an acceptable case definition used in the study? | |
|  | 7. Reliability and validity of study tool: Was the study instrument that measured the parameter of interest show to have reliability and validity? | |
|  | 8. Methods of data collection: Was the same mode of data collection used for all subjects? | |
|  | 9. Prevalence period: Was the length of the prevalence period for the parameter of interest appropriate? | |
|  | 10. Numerators and denominators: Were the numerator(s) and denominator(s) for the parameter of interest appropriate? |  |
|  | **The overall risk of bias scored based on the number of high risk of bias per study: low risk (≤2), moderate risk (3–4), and high risk (≥5).** | |
